# Management of Labor and Anesthesia in a Patient With a History of Spontaneous Intracranial Hypotension: A Case Report With Literature Review

**DOI:** 10.7759/cureus.52254

**Published:** 2024-01-14

**Authors:** Mayuko Nonaka, Shinichi Inomata

**Affiliations:** 1 Department of Anesthesiology, University of Tsukuba Hospital, Tsukuba, JPN; 2 Department of Anesthesiology, Division of Clinical Medicine, Institute of Medicine, University of Tsukuba, Tsukuba, JPN

**Keywords:** cesarean section, delivery, general anesthesia, pregnant, sih, spontaneous intracranial hypotension

## Abstract

Spontaneous intracranial hypotension (SIH) is a rare disorder characterized by continuous or intermittent cerebrospinal fluid (CSF) leakage from the CSF cavity, which causes symptoms such as headache or neck pain upon standing. However, no well-established measures concerning the type of delivery and anesthesia for pregnant women with a history of SIH have been reported. A woman had developed SIH 9 years earlier from lifting luggage into an overhead bin with stretching movements, which required continuous saline epidural infusion for recovery. Upon the patient’s pregnancy at the age of 35 years, although an elective cesarean section (CS) under general anesthesia was planned to avoid SIH recurrence, the patient had an emergency CS at 36 weeks. Since there is no prescribed method of delivery and anesthetic management for patients with a history of SIH, it is important to plan and adapt a treatment strategy based on the patient’s wishes and the institution’s protocols. As a sidenote, we reviewed the available literature regarding the type of delivery and anesthesia for pregnant women with a history of SIH.

## Introduction

Spontaneous intracranial hypotension (SIH) (International Classification of Headache Disorders, 3rd Edition) is characterized by continuous or intermittent cerebrospinal fluid (CSF) leakage from the CSF cavity resulting in symptoms, such as headache or neck pain on standing, dizziness, and tinnitus [1-3]. Although these symptoms are similar to those of low CSF pressure, SIH often lacks any obvious signs of trauma or other causes, and the CSF pressure might remain within the normal range. Few papers have reported the outcomes in pregnant patients with a history of SIH. This report describes a case of delivery in a woman with a history of SIH 9 years ago from lifting luggage into an overhead bin with stretching movements, the recovery for which required continuous epidural saline infusion. Moreover, we reviewed the available literature regarding the anesthetic management of pregnant patients with a history of SIH. Written informed consent was obtained from the patient for the publication of this case report.

## Case presentation

The patient was a 35-year-old woman with a height of 157 cm. Nine years earlier, at the age of 26 years, the patient developed a headache that worsened on standing and improved upon lying down after stretching to lift heavy luggage onto a shelf while working as a flight cabin attendant. Her symptoms did not improve, and she gradually became bedridden. She was admitted to our hospital after 8 days for examination and treatment. Upon admission, physical examination revealed no signs of meningeal irritation or neurological deficits; further, migraine-suggestive prodromal symptoms were absent. She did not completely recover after receiving nonsteroidal anti-inflammatory drugs.

**Figure 1:**
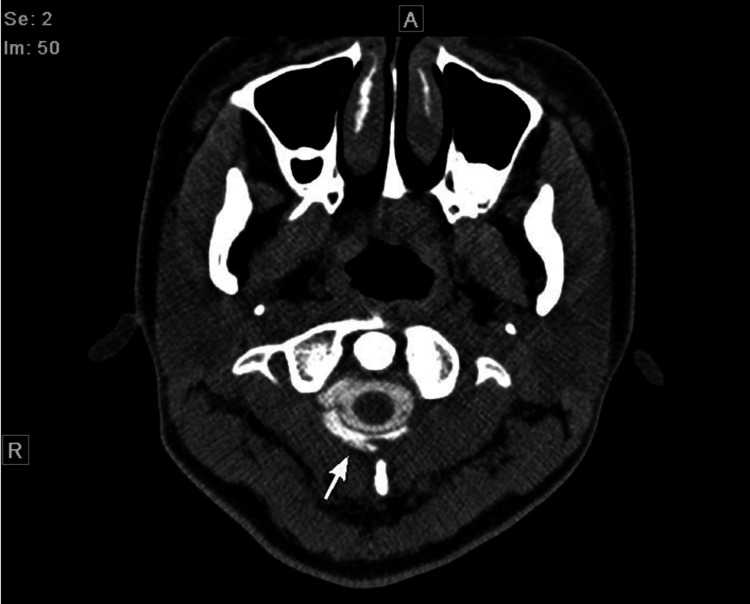
Contrast-enhanced computed tomography of the spinal cord shows extravasation of contrast into the epidural space

At the initial examination, the patient’s CSF pressure was normal (75 mmH2O {> 60 mmH2O}). However, contrast-enhanced computed tomography (CECT) of the spinal cord revealed CSF leakage from the cervical spine to T11; the patient was diagnosed with SIH (Figure 1).

**Figure 2:**
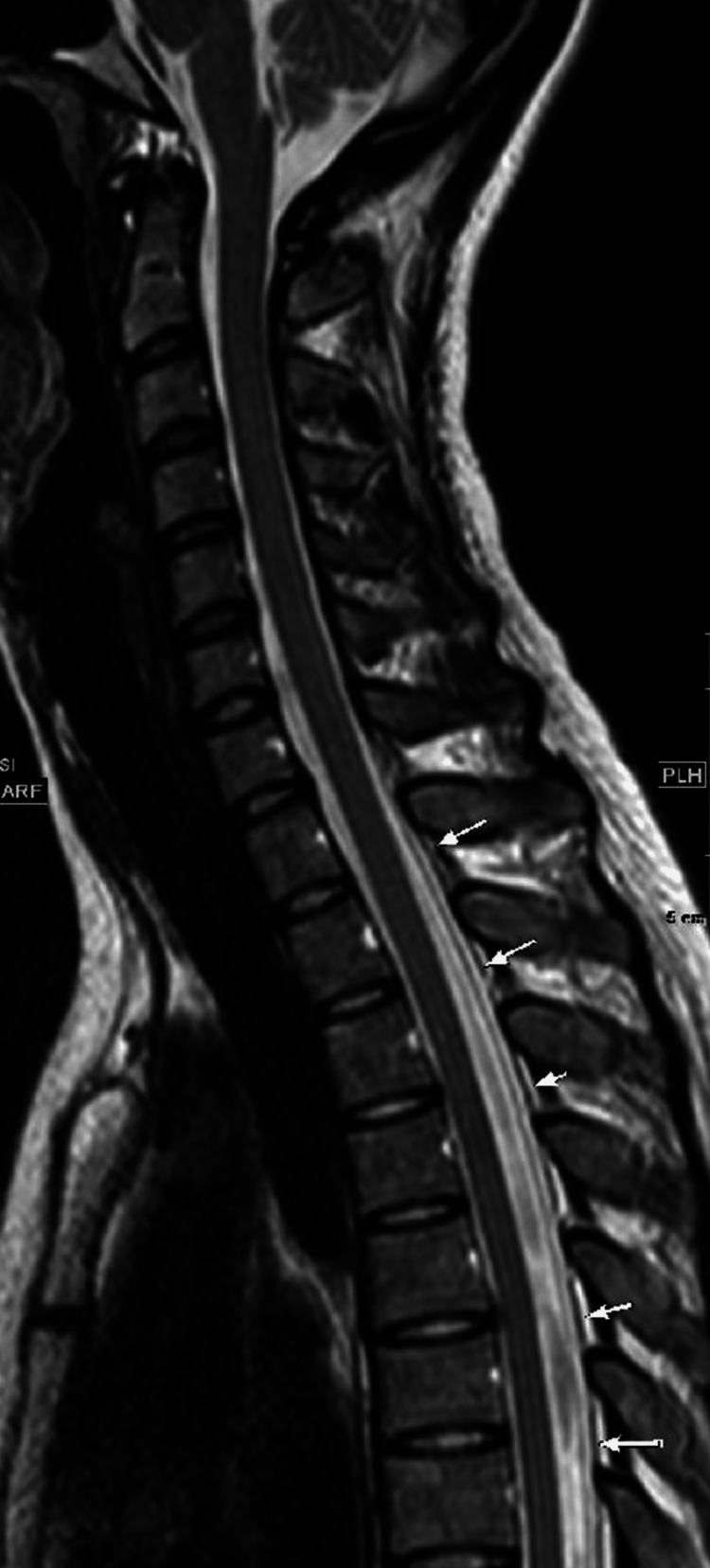
Magnetic resonance imaging myelography sagittal T2 shows fluid accumulation in the epidural space around T1–7

Magnetic resonance imaging (MRI) myelography revealed fluid accumulation in the epidural region around T1-7; however, the site of CSF leakage could not be identified (Figure 2).

Since the patient's symptoms had not improved with supplemental fluids and bed rest, she underwent continuous epidural saline infusion at the L1-2 level, which was selected to avoid accidental puncture of the dura mater, 3 weeks after admission. Although her symptoms mildly improved, the prolonged sitting and standing time and activities of daily living (ADL) did not improve. Her symptoms and ADL gradually improved upon switching the catheter to the T2/3 level 6 weeks after admission. The catheter was removed 2 months after admission, and the patient was discharged from the hospital. She entered nursing school and started working as a nurse, with the final medical examination being conducted 3 years after discharge. Her attending physician advised her to avoid excessive stress on the lumbar region and stretching movements to prevent the recurrence of SIH.

At the age of 35 years, the patient became pregnant with her first child. At a gestation period of 33 weeks and 1 day, she participated in an anesthesia consultation regarding the delivery and anesthesia methods. After discussions with her obstetrician, dura mater protection was prioritized, and delivery via general anesthesia and cesarean section (CS) was planned. At a gestation period of 36 weeks and 1 day, the patient presented with elevated blood pressure and increased urinary protein levels and was admitted to the hospital with severe preeclampsia. At a gestation period of 36 weeks and 3 days, she underwent an emergency CS under general anesthesia for severe preeclampsia. Anesthesia was rapidly induced using 130 mg propofol, 50 μg fentanyl, and 70 mg rocuronium. Anesthesia was maintained using 1.5% sevoflurane in oxygen until delivery, with propofol and remifentanil administered after delivery. Fentanyl was administered to avoid excessive blood pressure elevation and bucking. Postoperatively, we provided patient-controlled analgesia with fentanyl and regular acetaminophen administration. The newborn weighed 2497 g; had an Apgar score of 7 and 9 at 1 and 5 minutes, respectively, and an umbilical artery pH of 7.228; and did not require other treatments such as bag and mask ventilation. The patient has reported no signs of recurrence of headaches since the delivery.

## Discussion

According to the International Classification of Headache Disorders, the diagnostic criteria for SIH are based on the symptoms and CSF drainage as confirmed using MRI or CECT [3]. SIH management primarily involves rest and the administration of fluids, caffeine, and analgesics. When symptoms do not improve, an epidural blood patch is performed [1]. Moreover, in intractable cases, fibrin glue patches [4], dextran and steroid injections [5], and continuous epidural saline infusions [6] to the site of CSF leakage have been described as effective treatment modalities.

There can be three types of options under the anesthetic management of this case: vaginal delivery, CS under regional anesthesia, and CS under general anesthesia. Generally speaking, vaginal delivery is the most common mode of delivery, followed by CS under regional anesthesia and CS under general anesthesia. Our patient underwent a CS under general anesthesia, however, because Valsalva maneuver (straining by vaginal delivery) and CS under regional anesthesia both may take a risk of recurrence of SIH.

The Valsalva maneuver may cause CSF leakage in patients with a weak dura mater [2]. Although vaginal delivery is not contraindicated in pregnant women with a history of SIH [7], SIH can be caused by the Valsalva maneuver even without epidural or spinal anesthesia or dural puncture during pregnancy [8].

Only a few reports have described the outcomes in pregnant patients with a history of SIH. A literature search of Pubmed, Cochrane Library, and Embase databases from 1980 to 2022 using the following search terms: “spontaneous intracranial hypotension,” “cerebrospinal fluid,” and “pregnancy” yielded 13 reports of SIH development during pregnancy and delivery. Table 1 summarizes the patient characteristics and the delivery and anesthesia methods in these patients [5,9-15].

**Table 1:** Literature review of SIH cases during pregnancy G: gravidity, P: Parity, N/A: Not applicable, SIH: spontaneous intracranial hypotension, CS: cesarean section, MRI: magnetic resonance imaging, SVD: spontaneous vaginal delivery, CT: computed tomography, EBP: epidural blood patch

Author	Age	G	P	Triggering factors	Time of onset	Main symptom	Diagnostic imaging	Treatment	Type of delivery	Type of anesthesia	Note
Bel [5]	39	2	1	Mild exercise	32 weeks	Occipital headache	MRI	Oral analgesics and bed rest, oral dexamethasone, lumbar epidural injection of dextran-40, and paramethazone.	SVD at term	Epidural analgesia	
Asakura [9]	37	3	2	N/A	8 weeks	Orthostatic headache	MRI	Hydration, oral analgesics, and bed rest	SVD at 39 weeks	No anesthesia	
Singh [10]	33	2	1	N/A	30 weeks	Postural headache	MRI	Oral analgesics and bed rest, EBP	SVD at term	No anesthesia	
McGrath [11]	21	1	0	N/A	15 weeks	Intermittent frontal headache	MRI	EBP	SVD at term	N/A	
McGrath [11]	26	2	1	N/A	16 weeks	Intermittent frontal headache	MRI	Hydration, oral analgesics and bed rest, EBP	SVD at term	N/A	
Hashmi [12]	39	2	1	N/A	10 weeks	Occipital headache	CT	Hydration, oral analgesics and bed rest, caffeine, prednisone	N/A	N/A	
Grange [13]	30	3	2	N/A	28 weeks	Orthostatic headache	MRI	Oral analgesics, EBPs	CS at 36 weeks	Spinal anesthesia	
Ferrante [14]	38	4	3	Hyperemesis	16 weeks	Orthostatic headache	N/A	Hydration, oral analgesics and bed rest, EBP	SVD at 38 weeks	N/A	
Ferrante [14]	36	3	2	Frequent coughing fits	17 weeks	Orthostatic headache	MRI	Hydration, oral analgesics and bed rest, EBP	CS at 38 weeks	N/A	CS was performed at the request of the patient
Ferrante [14]	32	1	0	N/A	15 weeks	Orthostatic headache	MRI	Hydration, oral analgesics, and bed rest	SVD at 38 weeks	N/A	
Ferrante [14]	35	1	0	Hyperemesis	14 weeks	Orthostatic headache	MRI	Hydration, bed rest, EBP	CS at 37 weeks	N/A	CS was performed at the request of the patient
Ferrante [14]	35	2	1	Prolonged neck hyperflexion	6 weeks	Orthostatic headache	MRI	Hydration, bed rest, EBP	CS at 37 weeks	N/A	CS was performed at the request of the patient
Reihani [15]	32	2	1	N/A	34 weeks	Orthostatic headache	MRI	Oral analgesics, hydration, bed rest, caffeine	CS	N/A	CS was performed at the request of the doctor

Among them, McGrath et al. described two patients who developed SIH during pregnancy and received epidural blood patches. Both patients underwent vaginal delivery without SIH recurrence [11]. Ferrante et al. described five patients who developed SIH during pregnancy and delivered successfully following treatment; among them, two delivered vaginally while three underwent CS [14]. These reports indicated successful delivery following treatment for SIH without its recurrence; however, the number of reported cases is small (n = 12), and the anesthesia method was only described in two cases.

CS under regional anesthesia could possibly damage the dura mater. The mechanism underlying the improvement of SIH symptoms by saline infusion into the epidural space may include the prevention of CSF leakage through the application of continuous pressure at the site of the leakage and maintenance of the CSF pressure and volume through pressure on the dura mater. However, catheter placement with a continuous infusion into the epidural space and frequent epidural blocks can cause inflammation and adhesions in the epidural space [16]. Further, the epidural space in pregnant women is narrowed by the development of venous plexus and edema of the connective tissue [17]. However, we could not determine whether the epidural space had become adherent or narrowed in our patient. The patient was considered to have a relatively high risk of dural puncture using the epidural needle. Although the rate of dural puncture using Touhy needles during epidural anesthesia is usually only 0.8%, the rate of headache following dural puncture could be as high as 81% [18]. Although spinal anesthesia is considered the standard anesthesia protocol for CS, post-dural-puncture headache (PDPH) has been reported in 0.8% of cases even with the use of a 25-G pencil-point needle at our hospital [19].

Our patient considered it the most important thing to protect dura mater as she desperately wishes to avoid the risk of recurrence of SIH. She developed SIH at the age of 26 years and presented with chronic symptoms, which impeded her social life. Since the onset of SIH had been triggered by a stretching action involving lifting and extension, she was asked to minimize stretching movements in her daily life [20]. Accordingly, there were concerns regarding the recurrence of SIH due to the Valsalva maneuver during vaginal delivery, dural damage, or PDPH due attributed to regional anesthesia.

There were three factors considered when deciding the type of delivery and anesthesia. One was that we had the capability to treat neonates immediately after being delivered by CS under regional anesthesia (not all hospitals have neonatologists standing by to handle them with special measures). Another was that there was a consensus among all of our medical staff members that we should respect the patient's wishes the most. The other was that all the departments involved in this case considered the patient’s requests with her background reasonable from the medical perspective as well.

In our hospital, general anesthesia is performed in patients who cannot undergo regional anesthesia during CS or in cases of emergency CS. Additionally, the neonatologists' backup will facilitate the use of general anesthesia.

Accordingly, we discussed the various options for the delivery (natural vaginal delivery or CS) and anesthesia method (regional or general anesthesia) for the patient among the departments of anesthesiology, obstetrics, and neonatology and concluded that CS under general anesthesia would be the best option. Therefore, with the patient's wishes included, we finalized the performance of CS under general anesthesia.

The baby had an Apgar score of 7 at 1 minute owing to the anesthetic effect; however, it quickly recovered and the baby was discharged without prolongation of hospital stay or complications. The patient was satisfied after the operation and did not present any anesthesia-related or obstetric complications, or SIH recurrence

No well-established measures concerning the type of delivery and anesthesia for pregnant women with a history of SIH have been reported. Therefore, treatment needs to be adapted to the patient's wishes and institutional protocols with cooperation and communication among the departments of anesthesiology, obstetrics, and neonatology. Further reports are warranted to provide extended details regarding anesthesia methods for pregnancy and delivery in patients with a history of SIH.

## Conclusions

Despite its most uncommon anesthetic management of the three types of options in delivery of this case: vaginal delivery, CS under regional anesthesia, and CS under general anesthesia, the pregnant woman with a history of SIH underwent a CS under general anesthesia. This is completely to eliminate the risk of recurrence of SIH (both vaginal delivery and CS under regional anesthesia may cause recurrence of SIH), considering the respect for the patient's wishes not to damage dura mater, their reasonability and feasibility based on the level of medical support equipped in the institution. Since there have been no well-established measures regarding the types of delivery and anesthesia for pregnant women with a history of SIH, it is absolutely necessary to discuss and decide the types of delivery and anesthesia with patients and institutional protocols with cooperation and communication among the departments of anesthesiology, obstetrics, and neonatology in the perinatal period.
